# *In vivo* assessment of buparvaquone resistant *Theileria annulata* populations: genetic structure, transmission dynamics, drug susceptibility and pharmacokinetics

**DOI:** 10.1371/journal.pone.0334332

**Published:** 2025-10-15

**Authors:** Selin Hacilarlioglu, Huseyin Bilgin Bilgic, Cengiz Gokbulut, Serkan Bakirci, Ayca Aksulu, Brian Shiels, Andrew Tait, Onur Kose, Metin Pekagirbas, William Weir, Dilek Aksit, Tulin Karagenc

**Affiliations:** 1 Department of Parasitology, Faculty of Veterinary Medicine, Aydın Adnan Menderes University, Işıklı, Aydın, Türkiye; 2 Department of Pharmacology, Faculty of Medicine, Balıkesir University, Balıkesir, Türkiye; 3 School of Biodiversity, One Health and Veterinary Medicine, College of Medicine, Veterinary and Life Sciences, University of Glasgow, Bearsden Road, Glasgow, United Kingdom; 4 Department of Parasitology, Faculty of Veterinary Medicine, Burdur Mehmet Akif Ersoy University, Burdur, Türkiye; 5 Department of Forensic Sciences, Faculty of Engineering and Natural Sciences, Kütahya Health Sciences University, Kütahya, Türkiye; Guru Angad Dev Veterinary and Animal Sciences University (GADVASU), INDIA

## Abstract

Tropical theileriosis, caused by the protozoan parasite *Theileria annulata* and transmitted by several species of ixodid ticks of the genus *Hyalomma,* is an economically important disease of bovines. Concerningly, studies conducted in recent years have shown an increase in the rate of failure when using the primary drug of treatment, buparvaquone (BPQ), particularly in infection caused by *T. annulata* populations bearing V135A and P253S mutations on the *Cytochrome b* (*Cyto b*) gene of the parasite mitochondrial genome. The aim of this study was to demonstrate the relationship between BPQ-resistance and V135A and P253S mutations utilising an *in vivo* experimental set-up and to assess the tick transmissibility of drug-resistant populations. Additionally, the pharmacokinetics of BPQ in healthy and infected calves were compared to evaluate any relationship between plasma drug concentration and treatment failure. The study results demonstrated that, despite four consecutive BPQ treatments, animals infected with the resistant isolates exhibited more severe clinical signs, including longer periods of pyrexia, longer periods of schizont and piroplasm parasitemia, and the death of one animal. In addition, 3-(4,5-dimethylthiazol--yl)-2,5-diphenyltetrazolium bromide (MTT) analyses showed that all cell lines derived from animals infected with the mutant genotypes exhibited resistance to high BPQ concentrations. Unexpectedly, despite substantial calf-to-calf variation during the experiment, the genetic structure of the parasite population remained largely unchanged and no strong evidence for a major genotypic shift was detected. Plasma BPQ levels were similar in all groups tested. There was no association between plasma concentrations of BPQ and parasitological or clinical response to treatment. Live parasitaemia was observed even at high plasma BPQ levels in animals infected with resistant isolates. Significantly, drug resistant parasite populations harbouring either V135A or P253S mutations was transferred between the host and vector ticks, indicating the potential for resistant parasites to be transmitted from cattle in the field, thereby facilitating their maintenance in natural populations.

## Introduction

Tropical theileriosis is a tick-borne disease of cattle caused by the protozoan parasite *T. annulata.* The disease is responsible for serious economic losses due to high morbidity and mortality of infected animals across the Middle East and Asia, including North Africa, Southern Europe, India and Türkiye [1]. Strategies used to control tropical theileriosis include (i) drug treatment of infected animals, (ii) vaccination with attenuated cell lines, (iii) tick control and (iv) development of disease-resistant breeds. Of these regimes, the most widely used in endemic areas is the treatment of infected animals with BPQ, a hydroxynaphthoquinone compound. However, there are ongoing concerns over the effectiveness of BPQ due to a considerable increase in the number of reports of treatment failure from various countries since 2010 [2–6]. While details of the mechanism of action of BPQ are not yet fully established, the drug acts like atovaquone, a derivative of 2-hydroxynaphthoquinone [7]. Atovaquone, known for its broad-spectrum activity against *Plasmodium* spp., *Pneumocystis carinii*, *Babesia* spp., and *Toxoplasma gondii* [8–10], exerts its effect by binding to the oxidation site of ubiquinone on *Cyto b* which in turn leads to the collapse of the mitochondrial membrane [11,12]. Single and multiple nucleotide mutations in the *Cyto b* gene have been associated with atovaquone treatment failure in *Plasmodium falciparum, P. berghei, Babesia gibsoni* and *T. gondii* [12–14]. Nucleotide polymorphisms in the *Cyto b* gene of *T. annulata* have also been detected in parasite isolates derived from cases of treatment failure and are associated with resistance to BPQ [3–5,7]. In line with these observations, we have previously identified two mutations conferring amino acid substitution, *viz*. V135A and P253S, at the putative ubiquinone-binding sites between 116–144 (Q_O1_) and 242–286 (Q_O2_) regions of the predicted protein [6]. Both these mutations, appear to play a crucial role in the development of BPQ resistance [6]. The presence of mutation P253S within the Q_O2_ binding region was reported previously for *T. annulata* populations isolated in other endemic countries [3–5,7]. More recent studies have shown that BPQ-resistant parasite infected cells generated through prolonged *in vitro* exposure to BPQ harbour the predicted V135A and P253S mutations, and others, in the *Cyto b*, providing further support for an altered cytochrome b structure in the development of resistance to BPQ [15,16]. Nevertheless, to validate a potential role in resistance to drug treatment, the causal relationship between BPQ-resistance and the presence of candidate mutations in *Cyto b* needs to be demonstrated experimentally for natural infection *in vivo*. Additionally, understanding the impact of the gain of drug resistance on parasite fitness is of considerable importance, particularly regarding the transmissibility of the parasite to the host through the arthropod vector. The non-transmissibility of atovaquone-resistant *Plasmodium* isolates harbouring PbY268N/C, PfY268S, and PfV259L mutations in the *Cyto b* gene has been reported [17,18]. In contrast, atovaquone-resistant *P. chabaudi*, with mutations in the Q_O_ region of the *Cyto b* gene, is transmissible via *Anopheles stephensi* [19]. Until now, only one experimental study, conducted by Mhadhbi et al. (2010) [2] in Tunisia, has assessed BPQ resistance of *T. annulata in vivo* and the transmissibility of the resistant phenotype through ticks. While Mhadhbi et al. (2010) [2] showed that drug-resistant parasite phenotypes can be transferred to vector ticks, there is a need to demonstrate whether all related mutations in the *Cyto b* gene in drug resistant genotypes can be transmitted through ticks.

A number of factors, including drug pharmacokinetic properties and variable response across individuals, affect therapeutic efficacy of drugs [20,21]. Importantly, inadequate drug concentrations may allow resistant parasite genotypes to survive whilst removing susceptible ones [21]. Pharmacokinetic properties and dosage recommendations for BPQ have been determined mainly in uninfected or mildly infected animals [22,23]. However, active compound distribution may differ significantly in infected animals due to organ dysfunction [24,25]. Thus, it is essential to assess whether treatment failures are linked to altered BPQ plasma concentrations in severe cases of theileriosis.

In this study, we aimed to assess the response of BPQ-resistant *T. annulata* isolates possessing the V135A or P253S mutations in the *Cyto b* gene to drug treatment, *in vivo,* together with their tick transmissibility. Additionally, the pharmacokinetics of BPQ in healthy and infected calves were compared to determine the relationship between plasma drug concentration and treatment failure.

## Materials and methods

### Parasite material and experimental design

Two BPQ resistant and one sensitive *T. annulata* isolates were used in this study. Drug resistant *T. annulata* A10/BT and *T. annulata* A21/AT1 schizont-infected cell lines harbouring V135A and P253S mutations, respectively, were obtained from naturally infected cattle with a history of repeated BPQ treatments in the Aydın region of Türkiye, as described previously [6]. The *T. annulata* Ankara/279 schizont-infected cell line was used as the BPQ sensitive control isolate [26]. These isolates, derived from naturally infected cattle, represent both mutated drug-resistant and unmutated drug-susceptible parasite genotypes, thereby reflecting field conditions.

The study was approved by the institutional animal Ethics Committee of the Aydın Adnan Menderes University (Protocol number: 64583101/2013/018) and conducted according to national guidelines conforming to European Directive 2010/63/EU. The experimental animals used in the present study were obtained from a certified disease-free farm and all animals were screened to ensure the absence of *T. annulata* DNA by PCR [27] and parasite-specific antibodies by indirect fluorescent antibody test (IFAT) [28]. A total of 20 three-month-old Holstein calves were used in this study. Animals were kept in a special tick-free experimental facility throughout the study at Aydın Adnan Menderes University, Faculty of Veterinary Medicine which had been designed to minimise conditions that may result in suffering and/or distress. None of the experimental procedures required the use of anaesthesia or analgesia. Animals were fed with concentrate feed and wheat straw, and water was provided ad libitum throughout the study. Only research staff with a certificate of use of experimental animals were allowed to handle animals during each experiment.

Calves were routinely monitored from the day of infection (day 0) until the end of each experimental protocol. Rectal temperatures, prescapular lymph node enlargement and clinical signs of disease were monitored and recorded daily throughout the infection period. Blood samples were collected from the jugular vein three times a week into vacutainer tubes containing ethylenediaminetetraacetic acid (EDTA) for parasitological and haematological examination and lithium-heparin to establish schizont-infected cell lines *in vitro*, and for determining the pharmacokinetics of BPQ treatment.

**Fig 1:**
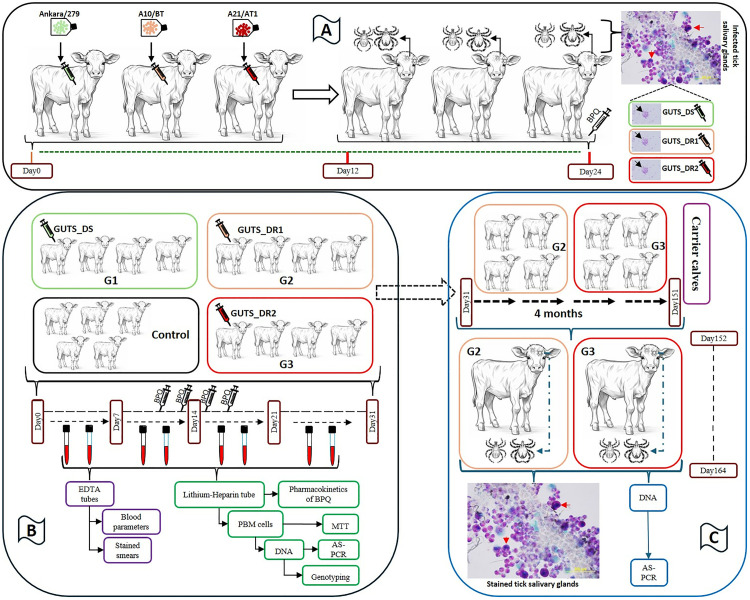
Schematic presentation showing the experimental work-flow. **(A)**. Infection of calves with two drug-resistant (A10/BT, A21/AT1) and one drug-sensitive (Ankara/279) cell line isolates of *T. annulata* to prepare sporozoite-infected ground-up tick stabilates (GUTS). GUTS_DS, GUTS_R1, and GUTS_R2 are designated for the stabilates representing Ankara/279, A10/BT and A21/AT1, respectively. Day 0 represents the day of infection for each group. Ticks were placed into the ear bags of calves after the first detection of piroplasms, and engorged ticks began to drop around Day 12 post-infection and were collected daily until Day 24, when buparvaquone treatment was administered. Red arrows indicate the infected tick’s salivary glands and black arrows indicate sporozoites extracted from each related tick’s salivary glands. **(B)**. Calves divided into three groups (G1, G2 and G3) and infected with GUTS from the two drug-resistant stabilates (GUTS_R1 and GUTS_R2) and one drug-sensitive stabilate (GUTS_DS) to assess the *in vivo* response of the calves to each isolate and to determine the pharmacokinetics BPQ in infected animals. The uninfected control group was kept to evaluate the pharmacokinetics of BPQ in healthy calves. **(C)**. Calves in groups G2 and G3 were kept in a tick-free experimental facility for about four months and fed *ad libitum*. Two calves from G2 and G3 were randomly selected to serve as carriers in transmission experiments. Red arrows indicate the infected tick salivary glands.

The experimental design incorporated the use of humane endpoints. These were based on our study design, treatment protocol used and published criteria for classifying *Theileria* disease severity [29]. Calves were considered to meet the endpoint criteria if they exhibited a HCT of <10%, live piroplasm (≥20%), and schizont parasitaemia for more than three weeks, along with clinical signs including pyrexia, interstitial pneumonia, dyspnoea, anorexia, weakness, coughing, petechial haemorrhages and jaundice. Any animal reaching this endpoint would be removed from the study and euthanased immediately in the designated slaughterhouse. The severity of infection was determined based on clinical, parasitological and haematological parameters [30]. Cases were classified as severe if the body temperature was ≥ 39.5°C for at least six consecutive days, and/or haematocrit (HCT) was < 20%. Cases with fever for less than three days, and a non-significant decrease in HCT level (≥26%) were considered to be mild infections; cases that fell between these severe and mild parameters were designated moderate infections. Animals were treated with intramuscular administration of BPQ (Butalex, 50 mg/mL, Intervet, Istanbul) at a dose of 2.5 mg/kg body weight when necessary. A schematic representation of the experimental work-flow is provided in Fig 1.

Initially, three calves were individually infected with *T. annulata* A10/BT, *T. annulata* A21/AT1 and *T. annulata* Ankara/279 cell line isolates to allow the creation of sporozoite-infected ground-up tick stabilates (GUTS) of these isolates (Fig 1A). When all ticks were fed and dropped into collection earbags, three calves were treated with BPQ at day 24 post-infection (PI) and withdrawn from the experiment after clinical recovery. Subsequently, they were euthanased in the designated slaughterhouse to prevent the spread of the disease. Euthanasia was carried out in a standard manner by jugular exsanguination according to FAO guidelines [31]. No calves reached the defined humane endpoint in this study. The nymphal stage of *Hyalomma excavatum* ticks used in this study were provided from the tick rearing unit at the Department of Parasitology, Faculty of Veterinary Medicine, Aydın Adnan Menderes University.

An experimental model was designed to assess the *in vivo* response of the calves to each isolate and to determine the pharmacokinetics of BPQ treatment in infected and healthy animals (Fig 1B). A total of 17 calves were used for *in vivo* experimental infection. The sample size was determined using the resource equation method [32] by calculating the “E” value. The measured resource equation resulted in an “E” value of 13, indicating that the sample size was within acceptable limits. Additionally, the sample size of this study aligns with that of previous *in vivo* studies [2,33], which have contributed valuable insights to the field. Briefly, twelve calves were divided into three groups (G1-3) two of which were infected with GUTS from putative drug-resistant isolates while one was infected with a known drug-sensitive isolates. Five animals were kept as the uninfected control group in order to determine drug pharmacokinetics in healthy calves. Calves in G1, G2 and G3 were routinely monitored from the day of infection (day 0) until the end of experiment (day 31 PI). Calves were treated with BPQ at an early stage of infection, i.e., as soon as the first parasite (piroplasm) was detected in stained blood smears. By initiating treatment at an early stage of infection, the study prioritised welfare of the animals. During the *in vivo* experiment, fever and the level of parasitaemia was monitored and the percentages of healthy and pyknotic degenerative piroplasms recorded after each BPQ treatment. Treatment with BPQ was repeated after 48 hours if fever or parasitaemia persisted in animals in G1-3. Calves did not receive any additional treatment other than BPQ until the end of the third drug treatment, as it might affect the pharmacokinetics of BPQ. Animals that did not respond to the third BPQ treatment within 48 hours received the fourth BPQ treatment together with symptomatic treatment of any clinical signs if present. Following recovery, calves in G1 and the control group were sent to the slaughterhouse.

Calves in G2 and G3 were kept in a tick-free experimental facility for about four months and fed *ad libitum*. Subsequently, two calves from G2 and G3 were randomly selected to serve as carriers in transmission experiments (Fig 1C). When all ticks were fed and dropped into earbags (day 12 PI), all calves in G2 and G3 were euthanased in the designated slaughterhouse to prevent the spread of resistant parasite populations. Calves in G1-3 and control group did not reach the defined humane endpoint during *in vivo* and transmission experiments.

### Preparation of sporozoite stabilates

Sporozoite-infected ground-up tick stabilates (GUTS) were prepared for *T. annulata* A10/BT, *T. annulata* A21/AT1 and *T. annulata* Ankara/279 cell line isolates by experimentally infecting calves with each isolate separately and feeding unfed *H. excavatum* nymphs during the acute phase of infection when piroplasm parasitaemia ranged between 0.3 to 4.0% (for A10/BT GUTS), 1.95–5.1% (for A21/AT1 GUTS) and 0.2–1.5% (for Ankara/279 GUTS), as described previously [34,35]. Briefly, calves were infected by subcutaneous inoculation of 10^7^ cells in front of the left prescapular lymph node. During the acute phase of infection, 4,000 unfed *H. excavatum* nymphs were placed in ear bags (2,000 nymphs on each ear). Following engorgement and moulting of ticks, the infection rate was evaluated by counting infected and uninfected acini cells of the tick salivary glands stained with methyl green/pyronine (MGP), as described by Walker et al. (1979) [36]. GUTS were prepared from infected adult ticks as described by Brown (1987) [35].

### Experimental infections of calves with resistant and susceptible sporozoite stabilates

Twelve calves were used for experimental infection with GUTS of the two resistant (A10/BT and A21/AT1) and susceptible (Ankara/279) *T. annulata* isolates. The animals were divided into three groups (G 1–3) such that the total body weight of animals in each group was equal. The calves each weighed approximately 94.6 kg, with a range of 70–118 kg (S1 Table). Doses of GUTS used for experimental infection were determined *in vitro* by stepwise titration of sporozoites infection of abundant peripheral blood mononuclear (PBM) cells in multi-well plates, as described by Wilkie et al. (2002) [37]. While animals in G1 and G2 were infected using GUTS at a dose of one tick equivalent (t.e.) of the susceptible Ankara/279 isolate and resistant A10/BT isolate, respectively, animals in G3 were infected with GUTS at a dose of 0.5 t.e. of the resistant A21/AT1 isolate.

The experimental infections were carried out as described previously [26,38] by subcutaneous injection of sporozoite stabilates slightly above the right prescapular lymph node. Calves were routinely monitored for 31 days from the day of infection (day 0). Animals with a rectal temperature above 39.5°C were considered to be febrile. Clinical signs of disease including pyrexia, depression, inappetence, weakness, lachrymation, petechial haemorrhages, prescapular lymph node enlargement on the side of the infection and anaemia were assessed and recorded throughout the infection period. Due to general effectiveness of BPQ in controlling piroplasm parasitaemia, additional parameters were used to monitor infection severity in calves in G1-3. The persistence of parasites (schizonts) in the lymph nodes, presence of viable piroplasms in circulating blood and body temperature ≥39.5°C 48 hours following treatment were considered to be indicative of severe infection. The immune status of the experimental animals was assessed by IFAT using schizont-stage antigens, as described in [28], on day 0 and on days 14 and 31 PI.

Giemsa-stained blood smears were prepared to detect the presence and the level of piroplasm parasitemia and haematological examination. The presence of schizonts and hyperplastic cells in the right prescapular lymph node was monitored by examining biopsy smears stained with Giemsa three times a week, starting on day 5 PI, then daily throughout the clinical reaction. Biopsy sampling was continued until schizonts were no longer detected for two consecutive biopsies, or until day 31 PI. Lymph node biopsy smears and thin blood smears were examined under light microscopy at 1,000 x magnification. Parasitaemia and schizont index were recorded as described by Darghouth et al. (1996) [38]. Briefly, the degree of parasitaemia was recorded as the percentage of infected red blood cells (RBCs) determined after counting 1,000 RBCs. The smear was recorded as negative if no piroplasms were detected over 50 microscopic fields. The level of schizont parasitaemia in lymph node smears was evaluated by examination of Giemsa-stained needle biopsy smears.

### Determination of the genetic structure of the *T. annulata* population following BPQ treatment

The effect of repeated BPQ treatments on parasite populations and the presence of parasite populations harbouring mutations were evaluated following each BPQ treatment using mini- and micro-satellite markers and allele-specific PCR (AS-PCR), respectively. Blood samples were collected into tubes containing either lithium-heparin (10 mL) or EDTA (5 mL), before each treatment and on day 31 PI. Blood samples containing lithium-heparin were used to establish schizont-infected cell lines from PBM cells *in vitro* [35]. Once the cell line established, cells were cryopreserved at early passage level [35] DNA was isolated from blood samples and established cell lines using the Promega Wizard genomic DNA extraction kit (Madison, WI, USA), following the manufacturer’s instructions, and stored at −20°C until use.

A total of five mini- and microsatellite markers (TS: 5, 20, 25 and TMSC: 75, 77) [39,40] were used to genotype DNA samples, using a previously described PCR-based protocol [40]. The size of amplicons representing the alleles of each mini- and micro-satellite marker was determined using high-resolution Spreadex gels (Elchrom Scientific™) with a resolution of 3 bp. Stained gels were visualised under UV light (254 nm) and VisionWorksLS (Versiyon 6.8) software (UVP EC3 Bio-Imaging system, USA) was used to determine allele size by direct comparison with a M3 marker (Elchrom Scientific). The number and frequency of alleles at each locus and expected heterozygosity within *T. annulata* populations following treatments in each group were assessed, as described previously [41]. Using this data, F_ST_ was calculated between the initial experimental time-point and each subsequent time-point in order to determine progressive levels of differentiation from the ‘before treatment’ parasite population. In isolates and blood samples obtained before each BPQ treatment, the presence of V135A or P253S mutations in parasite DNA were screened for by AS-PCR, utilising primers specifically designed to detect point mutations in the *Cyto b* gene of *T. annulata,* as described by Hacilarlioglu et al. (2023) [6].

### Susceptibility of *T. annulata* cell line isolates to buparvaquone

The susceptibility of cell lines obtained from experimentally infected calves following each BPQ treatment was determined by computing the proliferative index of *T. annulata* schizont-infected cell lines under various doses of BPQ using an MTT colourimetric assay [6]. Briefly, cells were exposed to BPQ in doses ranging from 0.4 to 1,000 ng/mL, in triplicate. The half-maximal inhibitory concentration (IC_50_) value was calculated for each cell line using optical density (OD) values, measured on an ELISA microplate reader (MultiscanGo, Thermo Fisher Scientific, USA) at 490 nm as the test wavelength and at 630 nm as the reference wavelength, with the aid of GraphPad Prism 5 software (GraphPad Software, La Jolla, CA, USA). The cut-off value for resistance of cell lines was adapted from our previous study [6].

### Feeding ticks on carrier animals infected with drug-resistant isolates of *T. annulata*

To examine the effect of repeated BPQ treatment on the transmission of drug-resistant parasite populations, ticks were fed on carrier animals infected with BPQ-resistant isolates (Fig 1C). One calf each from G2 (animal ID: 1343) and G3 (animal ID: 1135) was selected arbitrarily and fed *ad libitum* for four months to be used as carriers. A total of 4,000 un-fed *H. excavatum* nymphs (*Hex*IIAN) were then fed on the calves. Briefly, 1,000 ticks were placed in each ear bag until fully engorged. The engorged nymphs that dropped off the calves’ ears were collected and allowed to moult in an incubator (Memmert GmbH, IPP500, Germany) set at 80% relative humidity and 27°C. A total of 100 un-fed, infected adult ticks (50 males, 50 females) from each group (G2 and G3) were then crushed (5 ticks per pool-20 samples) with a sterile pestle in an Eppendorf tube under liquid nitrogen and used for DNA extraction, using a Promega Wizard genomic DNA extraction kit (Madison, WI, USA). Extracted DNA was used to detect the presence of resistant and/or susceptible parasite genotypes using AS-PCR, as described above. Salivary glands from a total of 20 ticks (10 male and 10 female) were dissected and stained with MGP to determine infection rates in tick acini cells, as described above.

### Pharmacokinetics of buparvaquone in uninfected and infected calves

In order to compare the pharmacokinetics of BPQ in infected calves (G1 - G3, n = 12) and healthy animals (pharmacokinetics control group, n = 5), heparinised blood samples (5 mL) were collected before drug administration and at 0.5, 1, 1.5, 2, 2.5, 3, 4, 6, 8, 12, 16, 24, 32, 48, 56, 72 and 96 hours post-treatment from each animal in both infection and control groups. BPQ (50 mg/mL, Butalex, Intervet, Istanbul) was administered intramuscularly at a dose of 2.5 mg/kg, as recommended by the supplier. Supplementary samples were collected from each animal at various time-points until 12 days after two additional administrations of BPQ, with 48 h intervals, to determine the subsequent kinetic parameters of BPQ in infected animals. Blood samples were centrifuged at 2,000 x *g* for 10 min, and plasma transferred to labelled plastic tubes. All plasma samples were stored at −20°C until used.

A stock solution (100 µg/mL) of the pure standard of BPQ (Product no: 32154, Vetranal® Supelco, Merck) was prepared using acetonitrile as solvent. This stock solution was diluted to prepare standard solutions of 0.5, 1, 5, 10, 20, and 50 µg/mL for calibration curves and for spiking drug-free plasma samples to determine validation parameters, such as recovery (%), linearity, intra- and inter-day variations, limit of detection (LOD) and limit of quantification (LOQ). An analytical simvastatin standard at 10 µg/mL (product no: S0650000, Sigma) was used as an internal standard.

Plasma concentrations of BPQ were determined by high-performance liquid chromatography (HPLC), using a photodiode array detector (PDA) after a solid phase extraction (SPE) procedure, as described previously [42], with minor modification. Briefly, drug-free plasma samples (1 mL) were spiked with BPQ standards to achieve the following final concentrations: 0, 0.01, 0.05, 0.1, 0.5 and 1.0 µg/mL. The spiked and experimental plasma samples were combined with 50 μl of the internal standard (simvastatin, 10 µg/mL) and then mixed with acetonitrile (1 mL with 2% glacial acetic acid). After shaking the tubes on a slow rotary mixer for 5 min, the solvent-sample mixture was centrifuged at 10,000 x *g* for 10 min. The supernatant was transferred to a C18 SPE cartridge (500 mg/6 mL, AccuBOND, Agilent, Waldron, Germany) previously conditioned with 3 mL methanol and 2 mL deionised water. The cartridge was washed with 2 mL water/methanol (4:1) and the samples were applied to the SPE cartridges. The cartridges were then dried under vacuum for 30 min. The analytes were eluted with 3 mL acetonitrile and concentrated to dryness at 45°C in a vacuum concentrator (Maxi-dry plus; Heto Lab. Equipment, Denmark). Reconstitution was performed with 250 µl of the mobile phase consisting of acetonitrile and ultrapure water with 1% acetic acid (74:26, v/v). After vortexing for 15 seconds, a 50 μl aliquot of this solution was injected directly into the chromatograph. The samples were processed on a computerised HPLC system (1260 series, Agilent, Waldron, Germany). The mobile phase was delivered at a flow rate of 1.2 mL/min. A nucleosil C_18_ analytical column (Zorbax Eclipse XDB-C18,4.6 x 250 mm, 5µl, Agilent, Waldron, Germany) with nucleosil C_18_ guard column at 40⁰C was used for analyses. The photodiode array detector was set to a wavelength of 252 nm.

### Data analysis

The Statistical Package for the Social Sciences (SPSS) for Windows Version 21 (SPSS Inc., Chicago, IL, USA) was used to analyse parameters such as packed cell volume (PCV), white blood cell (WBC), red blood cell (RBC) values and fever among the treatment groups (G1-3). The suitability of the parameters for tests requiring normally distributed values was evaluated using the Shapiro-Wilk (W) test. One-way Repeated Measures ANOVA was used to compare normally distributed parameters among the treatment groups. *p*-values **<* *0.05 were considered to be significant.

The number of alleles per marker per calf was determined at each experimental time-point and averaged across markers. This allowed expected heterozygosity, also known as gene diversity, to be calculated for each calf/timepoint/marker combination. The major allele was also determined and was defined as being the one corresponding to the most intense band on the gel image. In order to illustrate how the genetic composition of the parasite population changed over time, pair-wise F_ST_ values were calculated for timepoints AT1, AT2, AT3, AT4 and D31 with respect to the initial sample, BT, for each calf and this value was reported as the genetic distance between populations.

Plasma concentration versus time curves obtained after each treatment in individual animals were fitted using the WinNonlin software programme (version 5.2, Pharsight Corporation, Mountain View, CA, USA). Pharmacokinetic parameters for each animal were analysed using a non-compartmental model analysis for an extra-vascular drug administration. The maximum plasma concentration (Cmax) and time to reach maximum concentration (Tmax) were determined from the plotted concentration-time curve of each drug in each animal. The trapezoidal rule was used to calculate the area under the concentration-time curve (AUC) and the mean residence time (MRT) was calculated as follows:


MRT0→∞=AUMC0→∞ / AUC0→∞

The terminal half-life (T1/2λz) was calculated as follows:


T1/2λz=−ln(2) / λz

Where λz represents the first order rate constant associated with the terminal (log-linear) portion of the curve. Mean pharmacokinetic parameters (±SD) were compared between treatment groups using the one-way method ANOVA. All statistical analyses were performed using MINITAB for Windows (version 12.1, Minitab Inc., State College, PA, USA). Mean values were considered significantly different at *p* < 0.05.

## Results

### Clinical reaction of calves to infection

**Fig 2:**
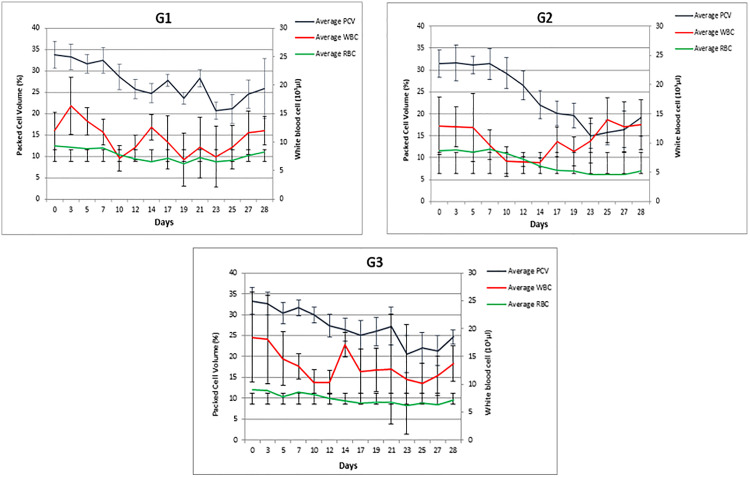
Haematological parameters of calves infected with BPQ-susceptible (G1) and BPQ-resistance (G2 and G3) *T. annulata* sporozoite stabilates. PCV: packed cell volume; WBC: white blood cells; RBC: Red blood cells.

**Fig 3:**
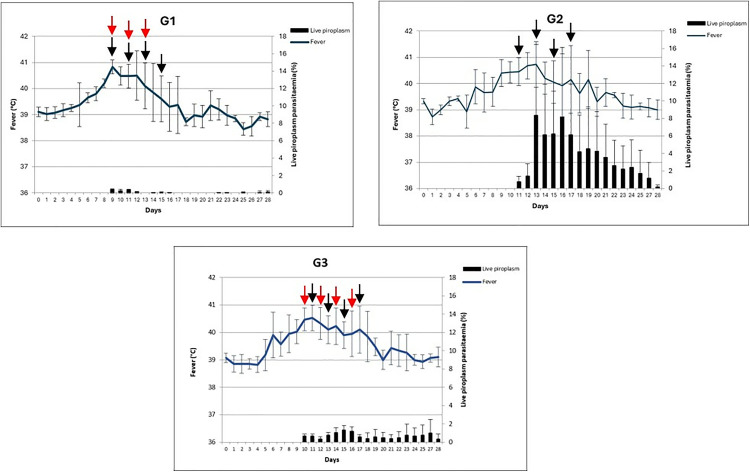
Clinical and parasitological responses in calves infected with BPQ-susceptible (G1) and BPQ-resistance (G2 and G3) *T. annulata* sporozoite stabilates. Red arrows represent the days of BPQ treatment applied to calves 1065 and 1135 in G1 and G3, respectively following infection. Black arrows represent the days of BPQ treatment applied to the remaining calves following infection.

The haematological, parasitic and febrile reactions of calves are given in Figs 2, 3 and in Table 1, respectively. All animals in each group developed clinical reactions to infection with varying intensities. While between-group differences in pyrexia were not significant, fever varied significantly during the course of infection in all groups, lasting for 6–12 days in calves infected with the drug-susceptible Ankara/279 isolate, 9–16 days in calves infected with the drug-resistant A10/BT isolate, and 8–15 days in calves infected with the drug-resistant A21/AT1 isolate (Table 1, Fig 2). Haematological values of animals also changed markedly. WBC values decreased significantly during the first three weeks of infection across all groups (see Table 1 and Fig 1). PCV also declined significantly over time in all groups, with reductions varying between 38.0% and 54.2% (Table 1, Fig 2). Significant differences were also observed between groups at specific time points. Calves infected with the drug-resistant A10/BT isolate had lower PCV values than those infected with the susceptible Ankara/279 isolate on days 17, 21 and 27 PI, and lower RBC values on days 17, 21, 23, 25 and 27 PI. In addition, differences were noted between A10/BT and A21/AT1-infected calves, with PCV significantly lower in the A10/BT group at day 27 PI and RBC lower in the A21/AT1 group at day 23 PI (Table 1).

**Table 1:** Clinical, haematological and parasitological parameters of experimentally infected animals.

Calf ID	Group 1 (G1)				Group 2 (G2)				Group 3 (G3)			
	1065	6859	9270	1344	2155	6857	1343	6816	0770	6825^X^	3674	1135
**Sporozoite stabilate (infection dose)**	Ankara/279 (1 t.e.)				A10/BT (1 t.e.)				A21/AT1 (0.5 t.e.)			
First onset of fever* (dpi)	6	6	6	5	3	3	6	9	6	5	6	10
**Duration of fever (Days)**	6	8	8	12	14	16	9	9	10	15	13	8
**Duration of fever after the first BPQ treatment (Day)**	2	4	4	8	8	10	3	6	4	8	7	7
**First onset/ duration of schizonts (dpi)**	5/ 9	5/ 9	5/ 9	5/ 8	6/ 23	5/16	6/ 15	5/ 14	8/ 12	6/ 13	6/ 15	6/ 15
**Detection of first piroplasm (dpi)**	9	9	9	9	11	11	11	11	11	11	11	10
**Maximum % of live parasitaemia (dpi)**	0.50 (9)	0.45 (9)	0.36 (11)	0.34 (9)	1.34 (15)	2.74 (15)	15.58 (13)	17.80 (16)	0.96 (11)	1.45 (15)	1.26 (15)	2.72 (27)
**Maximum % reduction in PCV (dpi)** ^a^	42.0 (23)	37.1 (25)	33.9 (25)	39.7 (25)	43.4 (23)	51.6 (23)	51.9 (23)	69.9 (27)	48.8 (23)	34.1 (14)	35.8 (23)	41.2 (27)
**Maximum % reduction in RBC (dpi)** ^b^	39.4 (19)	30.6 (25)	27.1 (14)	41.8 (23)	32.7 (17)	51.7 (21)	54.3 (23)	69.2 (27)	37.7 (23)	35.0 (17)	28.2 (19)	46.8 (27)
**Maximum % reduction in WBC (dpi)** ^a^	57.1 (19)	55.7 (10)	60.9 (10)	57.2 (19)	56.2 (23)	67.7 (23)	61.2 (10)	85.3 (14)	66.2 (12)	52.2 (10)	71.8 (23)	59.2 (25)
**Days of BPQ treatment applied**	9,11,13	9, 11, 13, 15			11, 13, 15, 17				11, 13, 15, 17			10,12,14,16

*: indicates measured body temperature above 39.5˚C; ^a^: indicates a significant reduction observed in PCV (*p* = 0.0001) and RBC (*p* = 0.0001) values over the time in G1, G2 and G3; ^b^: indicates a significant decrease in WBC values observed in the first three weeks (*p* = 0.022); PCV: packed cell volume; WBC: white blood cells; dpi: indicates days PI and t.e.: indicates tick equivalent dose. ^X^: indicates that the calf died on day 19 PI.

BPQ treatment was initiated after the detection of piroplasms in peripheral blood smears. In calves infected with the susceptible Ankara/279 isolate, three animals required four treatments at 48h intervals (from day 9, PI), while one animal received only three treatments. All calves infected with the resistant A10/BT and A21/AT1 isolates received four BPQ treatments at 48h intervals (from day 11, PI for A10/BT and day 10, PI for A21/AT1). Despite repeated administrations, fever persisted longer in resistant isolate infections and additional symptomatic treatments were necessary in some cases (Table 1, Fig 2).

Schizonts were detected in lymph node smears from day 5 PI onwards. The density of schizont-infected mononuclear cells was higher in calves infected with resistant isolates compared to those infected with the susceptible isolate (Table 1, Fig 3). Marked differences were also observed in piroplasm parasitaemia. In calves infected with the susceptible Ankara/279 isolate, parasites were eliminated within one week of BPQ treatment, except in one calf where low-level positivity persisted until day 28 PI (S2 Table). In contrast, in calves infected with the resistant A10/BT isolate, parasitaemia remained above 5% for 8–12 days, with values of 7.9–8.4% even three days after the fourth BPQ treatment (S2 Table). In one calf (1135) infected with the resistant A21/AT1, parasitaemia progressively increased after each treatment, reaching 2.72% on day 27 PI. Before reaching the experimental humane end-point, another calf (6825) died on day 19 PI despite four BPQ administrations and following additional symptomatic treatments given due to fever and persistent parasitaemia. In addition, piroplasm parasitaemia gradually increased in calf 1135 after each drug treatment with a live piroplasm level of 2.72% evident on day 27 PI (S2 Table). All infected animals developed antibodies against *T. annulata* by day 31 PI, as determined by IFAT, demonstrating an immune response to infection. These results validated, *in vivo*, the drug-resistant phenotype of the A10/BT and A21/AT1 sporozoite stabilates.

### Establishment of schizont-infected cell cultures

The number of isolates successfully cultivated as schizont-infected cell lines varied among calves in each group (S3 Table). In G1 (Ankara/279 sporozoite stabilate), cell lines were established up to the third BPQ treatment. Lines were obtained from all calves after the first treatment from three calves after the second, and from a single calf after the third. In G2 (resistant A10/BT GUTS), schizont-infected cell lines were successfully established at all time points until day 31 PI, except for two cases: one after the first treatment and another at day 31 PI. In G3 (resistant A21/AT1 GUTS), cell lines were obtained up to the fourth treatment (day 17 PI), with two exceptions: one after the first and one after the second treatment (S3 Table).

### Susceptibility of *T*. *annulata* isolates to repeated BPQ treatment

Results of the MTT analyses are given in Table 2. Cell line isolates obtained from animals in G1 before treatment were found to be sensitive to very low concentrations of BPQ with IC_50_ values ranging from 0.33 to 1.79 ng/mL. For isolates obtained from calves in G2 and G3, higher IC_50_ values under high BPQ concentrations were obtained, indicating the persistence of drug-resistance from the beginning to end of experimental infection (Table 2). Indeed, cell lines obtained from animals in G2 before treatment displayed elevated drug-resistance even at very high BPQ concentrations (average IC_50_ value of 150.23 ng/mL), and all isolates were still resistant to high drug concentrations (average IC_50_ value of 122.45 ng/mL) after the fourth drug treatment (AT4). In the isolates obtained from animals in G3 an increase in IC_50_ values was observed after each BPQ treatment (Table 2), indicating an increase in the resistance of the infected cell population to the drug.

**Table 2:** IC_50_ values of *T. annulata* schizont-infected cell lines obtained from animals infected with a susceptible (G1) and two resistant (G2 and G3) sporozoite stabilates.

Experimental groups		IC_50_ Values (ng/mL)				
		BT	AT1	AT2	AT3	AT4
**G1**	1065	0.33	1.47	16.38	–	–
	9270	1.79	12.17	–	–	–
	6859	0.96	12.17	10.02	–	–
	1344	0.80	5.34	15.31	–	–
**G2**	1343	107.4	–	64.47	82.31	141.9
	6857	192.3	99.90	57.60	86.99	81.40
	2155	228.0	223.5	185.6	125.3	134.9
	6816	73.23	51.78	185.3	102.3	131.6
**G3**	3674	10.20	40.27	50.26	85.70	102.8
	1135	17.60	–	11.66	10.52	86.46
	6825	10.37	14.69	24.21	32.01	28.57
	0770	17.81	31.05	–	40.78	52.77

BT: indicates *T. annulata* isolates prepared before drug treatment.

AT: indicates *T. annulata* isolates prepared after drug treatment.

AT1–4 represents the number of drug treatments.

### Presence of V135A and/or P253S mutations following repeated buparvaquone treatment

The effect of repeated BPQ treatments on the parasite populations of each experimental group was evaluated by screening cell line isolates along with blood samples obtained before and after each treatment for mutations in the *Cyto b* gene. Neither of the two mutations previously linked to drug resistance was detected in DNA derived from cell lines obtained from calves in control group G1 (S4 Table). The V135A mutation was detected only in cell lines obtained from calves in G2, and all lines also represented genotypes carrying unmutated *Cyto b*, except the cell lines obtained from calves 6857 and 2155. In these lines, the unmutated *Cyto b* parasite population was no longer detected for the line isolated after the fourth BPQ treatment and the line from day 31 (S4 Table). The mutation P253S was detected only in cell lines obtained from calves in G3, where all cell lines contained genotypes representing the mutated (P253S) and unmutated allele, except for the genotypes from the cell line isolated before treatment (calf 1135), which harboured only the unmutated allele.

The results of AS-PCR performed on blood samples were similar to those obtained from the cell lines except for the presence of both unmutated and mutated alleles in all samples collected from animals in G2 and G3. Only unmutated alleles were detected in blood samples collected from animals in G1 (S4 Table). The results confirm an association between previously identified mutations in *Cyto b* and resistance to BPQ*.*

### Genetic structure of parasite populations following treatment by genotyping

A total of five mini- and micro-satellite markers were used to investigate the genetic structure of the parasite populations under BPQ pressure. On average, two to six alleles at five different loci were detected in 77% of blood samples. The distribution of all alleles along with the number of genotypes detected before treatment, after each treatment and on day 31 PI in each group are given in S5–S7 Table. Although there were fluctuations in the parasite population of each calf during the experiment, particularly in G2 and G3 compared to the control group (G1), none of the groups showed a significant reduction in the number or complexity of genotypes following treatment (S5–S7 Table).

**Fig 4:**
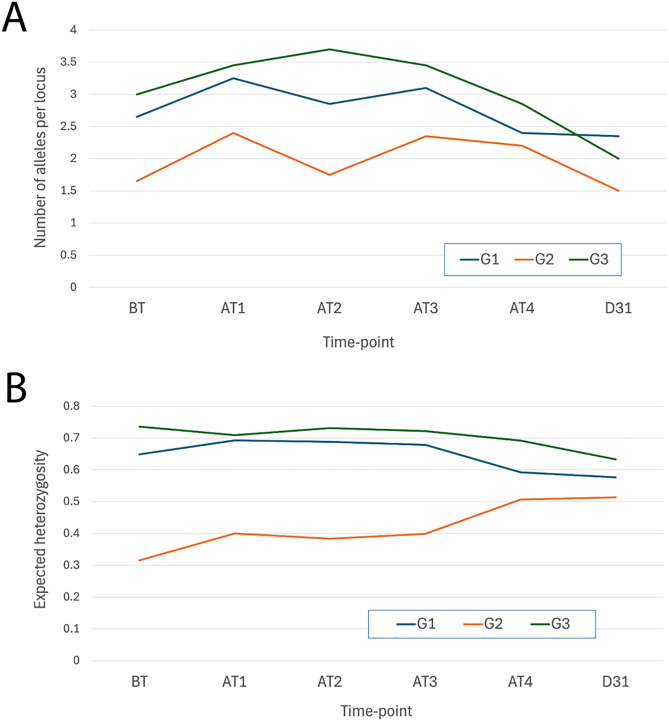
The average number of alleles present across loci for each of all groups (A) and the expected heterozygosity (B) at various time point throughout the study.

The data on the number of alleles and the expected heterozygosity (Fig 4) reflect the genetic diversity within each group. These findings indicate that (a) all groups contain mixed populations of *T. annulata*, (b) G3 has the highest diversity while G2 has the lowest, both in terms of the number of alleles present and heterozygosity, and (c) there is no strong evidence of genotype selection over the course of the experiment, as population complexity remains largely unchanged.

Additionally, the genetic distance analyses (Table 3) highlight intra-calf variation in the level of turn-over of parasite genotype. The control group (G1) shows that genotype turnover occurs at every time-point even in the absence of a drug-resistant parasite population. The most significant changes (BT → D31) were observed in one calf from G2 (calf 1143) and one from G3 (calf 1135), where an immediate genotype shift is evident at the last two time-points and after the first treatment which persisted throughout the experiment, respectively.

**Table 3:** The genetic distance between samples at various time points during the study.

Group	Calf	Time-point*					
		BT	AT1	AT2	AT3	AT4	D31
G1	1065	0.00	0.19	0.19	0.27	0.00	0.19
	9270	0.00	0.13	0.14	0.13	0.14	0.13
	6859	0.00	0.25	0.25	0.29	0.20	0.44
	1344	0.00	0.25	0.25	0.25	0.43	0.20
G2	6816	0.00	0.33	0.27	0.33	0.33	0.27
	1343	0.00	0.00	0.00	0.00	0.75	0.67
	2155	0.00	0.25	0.25	0.14	0.25	0.14
	6857	0.00	0.40	0.40	0.40	0.40	0.00
G3	770	0.00	0.00	0.07	0.07	0.21	0.56
	6825	0.00	0.11	0.24	0.25	0.15	0.00
	3674	0.00	0.38	0.31	0.31	0.38	0.42
	1135	0.00	0.76	0.71	0.72	0.69	0.71

*; Genetic distances relative to the before-treatment time-point are represented by shading, with values approaching 0 in yellow and those approaching 1 in green.

### Effect of repeated buparvaquone treatment on the transmission of drug-resistant parasite populations to ticks

The transmission rate of resistant (A10/BT and A21/AT1) populations to *H. excavatum* was evaluated by feeding un-fed nymphs on two carrier calves, one from G2 and the other from G3, following the fourth BPQ treatment. Ticks were not fed on a drug-susceptible G1 animal due to its well-known transmissibility which was demonstrated in our previous experiments at a rate of 97%. Microscopic examination of Giemsa-stained blood smears on the day of tick feeding indicated that the level of parasitaemia was 0.01% in calf 1343 (G2) and 0.3% in calf 1135 (G3). Data gathered from AS-PCR analyses demonstrated the presence of both unmutated and mutated parasites (harbouring mutations V135A and P253S) in calves 1343 and 1135. None of the acini cells were found to be infected with *T. annulata* sporozoites in salivary glands of ticks fed on calf 1343 (G2). Additionally, no parasite DNA was detected by AS-PCRs in these ticks.

All ticks fed on calf 1135 in G3 were found to be positive for *T. annulata* by MGP staining of the salivary gland and AS-PCR. The average infection rate in the salivary glands was 285 infected acini per tick. AS-PCR analyses demonstrated that 40% of the ticks harboured parasites carrying the P253S mutation, while another 40% were infected with an unmutated parasite *Cyto b* gene. The remaining 20% of ticks carried both the mutation and unmutated alleles.

### Pharmacokinetics of BPQ in uninfected and infected calves

Animals observed throughout the study showed no adverse clinical reaction following BPQ treatments. Prior to analysing experimental plasma samples, analytical methods for BPQ were validated using spiked plasma samples. The mean recovery of BPQ from the spiked plasma sample was 87.92 ± 6.65% and the LOD and LOQ for BPQ were found to be 0.003 µg/mL and 0.01 µg/mL, respectively.

**Fig 5:**
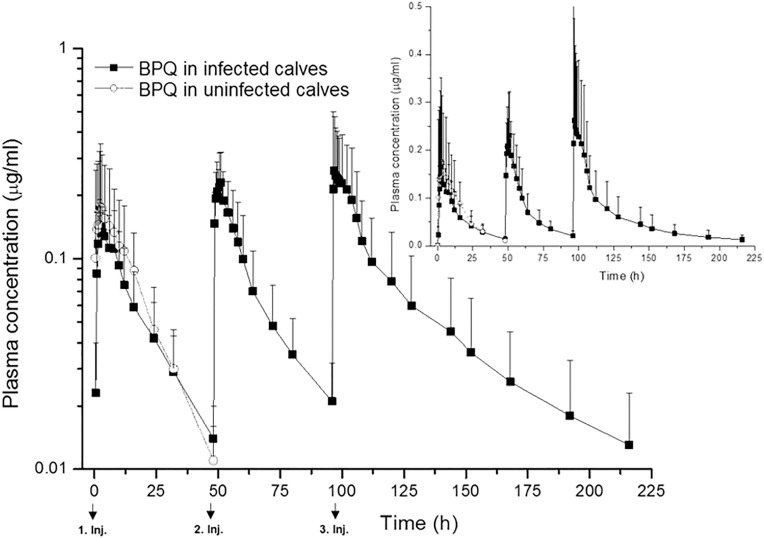
Semi-logarithmic plot of the mean (±SD) plasma concentration vs time curves of BPQ in uninfected calves following single injection and infected calves following three injections with 48 h intervals. The smaller graph is a linear plot of mean plasma concentration *vs.* time curves of BPQ.

Mean (±SD) pharmacokinetic parameters in uninfected and experimentally infected calves following intramuscular (2.5 mg/kg) administration of BPQ are given in Table 4. Mean plasma concentration *versus* time curves of BPQ in uninfected and experimentally infected calves are shown in Fig 5.

**Table 4:** Pharmacokinetic parameters of BPQ in uninfected and infected calves following intramuscular administration.

Kinetic Parameters	Uninfected Calves (n = 5)	Infected Calves (n = 10)		
	Single Injection	First Injection	Second Injection	Third Injection
**T**_**1/2λz**_ **(h)**	12.45 ± 2.44	13.82 ± 1.57^a^	14.32 ± 1.52	32.82 ± 8.46
**T**_**max**_ **(h)**	7.20 ± 5.76	3.50 ± 2.48	2.30 ± 0.95	2.30 ± 1.86
**C**_**max**_ **(µg/mL)**	0.21 ± 0.15	0.16 ± 0.05	0.26 ± 0.10	0.35 ± 0.27
**AUC**_**last**_ **(µg.h/mL)**	3.35 ± 1.88	2.56 ± 1.01^a^	3.48 ± 1.56	7.02 ± 4.78
**AUC**_**0→∞**_ **(µg.h/mL)**	3.43 ± 1.86	2.85 ± 1.11^a^	3.91 ± 1.74	7.23 ± 4.85
**AUMC**_**last**_	53.74 ± 26.36	39.68 ± 17.20^a^	50.08 ± 25.12	256.65 ± 207.37
**AUMC**_**0→∞**_	60.00 ± 20.08	59.32 ± 24.80^a^	79.53 ± 38.56	318.08 ± 246.87
**MRT**_**last**_ **(h)**	16.88 ± 4.36	15.33 ± 1.31^a^	14.09 ± 1.31	34.88 ± 8.90
**MRT**_**0→∞**_ **(h)**	18.95 ± 5.45	20.78 ± 2.39^a^	20.00 ± 2.18	42.20 ± 11.19

T_1/2λz_: terminal half-life, T_max_: time to reach peak plasma concentration, C_max_: peak plasma concentration, AUC_**0→∞**_: area under the curve, AUMC_**0→∞**_: area under the moment curve, MRT_**0→∞**_: mean residence time. Data refer to mean and standard deviations of measurements. Uninfected calves received a single dose of BPQ while infected calves received three doses separated by 48 hours at a dose of 2.5 mg⁄kg bodyweight. The kinetic parameters of BPQ in infected calves after first administration are significantly different (^a^ **p* *< 0.05) than those observed after second and third administrations.

Large inter-individual variations of the plasma concentrations and kinetic parameters within the group were observed from animals in both uninfected and experimentally infected groups (see Table 4, Fig 5). Although the uninfected group exhibited higher peak plasma concentrations, a greater area under the curve and a longer time to reach peak plasma concentration, these differences were not statistically significant between infected and uninfected groups (Table 4). Furthermore, despite a significantly higher peak plasma concentration in experimentally infected animals after the second dose compared to the first one, there was no significant differences in other kinetic parameters. Although, following the third dose of drug in infected animals, significantly higher plasma concentrations and plasma residence times were observed.

The effect of drug on the viability of the piroplasms in each group was compared by assessing the cumulative ratio of degenerate/dead piroplasms between days 9 and 28 PI (Table 5). Notably, the mean ratio of degenerate/dead piroplasms to total piroplasms in G1 animals at day 18 post drug-treatment was statistically higher (*p* < 0.005) than the ratio in G2 and G3 animals (Table 5). However, the amount of drug in systemic circulation, did not correlate with the ratio of degenerate/dead piroplasms. Despite high plasma concentrations of BPQ, both live piroplasms and schizonts continued to be detected in groups G2 and G3 infected with drug resistant isolates (Table 5).

**Table 5:** Cumulative ratio of degenerate/dead piroplasms (DDP) between days 9 and 28 PI in animals infected with BPQ-susceptible (G1) and BPQ–resistant (G2 and G3) isolates.

Experimental groups	Piroplasms (9–28 days PI)			
	DDP	Live	% of DDP	% of Mean DDP
**Calves in G1**				
**1065**	104	44	70.27	*70.89 ± 13.93
**9270**	16	15	51.61	
**6859**	120	24	83.33	
**1344**	47	13	78.33	
**Calves in G2**				
**1343**	614	2160	22.13	26.14 ± 3.51
**6857**	73	216	25.26	
**2155**	60	136	30.61	
**6816**	804	2225	26.54	
**Calves in G3**				
**3674**	78	116	40.21	34.31 ± 5.84
**1135**	184	386	32.28	
**6825**	39	105	27.08	
**0770**	90	149	37.66	

*: indicates statistical significance (*p* < 0.005). The mean ratio of degenerate/dead piroplasms in total piroplasms observed in animals (G1) infected with the sensitive isolate was statistically significant (*p* < 0.005) compared to those observed in animals (G2 and G3) infected with resistant strains.

## Discussion

Since the late 1980s, the hydroxynaphthoquinone compound BPQ has been used for the treatment of *T. annulata*-infected animals in endemic regions across the world. While new agents to combat tropical theileriosis are under investigation, and despite some promising data [15,43], there is currently no alternative treatment option. Consequently, BPQ remains the only drug to effectively manage the disease in the field. It is known that persistent exposure of existing parasite populations to a given drug can potentially result in the development of resistance [44]. There is extensive use of BPQ in regions where tropical theileriosis is endemic and strong evidence to indicate that resistance has developed in field populations of *T. annulata* [2,6]. *In vitro* studies [6] also indicate that genetic mutations identified at the potential binding sites of BPQ in *Cyto b* gene, *viz.* V135A and P253S, are involved in this process and predict positive selection of BPQ-resistant *T. annulata* genotypes. Together these studies indicate an association between a P253S substitution within the Q_O2_ region of cytochrome b and resistance to BPQ [3–5,7]. Nevertheless, a causal association between resistance to BPQ and the presence of V135A and P253S mutations in the *Cyto b* gene remained to be demonstrated *in vivo*. In the present study, we demonstrate this association: parasite populations with these substitutions correlated with more severe and persistent disease outcomes following repeated treatments with BPQ. While differences between an innate immune response between groups cannot be fully discounted by the study, to achieve the results obtained, animals infected with parasite genotypes lacking the detected mutations in *Cyto b* would be required to show a greater innate or acquired immune response against infection. Although limited to detection of antibody, there was no major difference between experimental groups in the level of a protective acquired immune response, over the time frame of the study. Thus, the results indicate that parasite strains which possess these mutations are resistant to drug treatment *in vivo* and so may be subject to positive selection in the field.

In the routine treatment protocol, a single dose of BPQ (2.5 mg/kg) typically cures infected animals within days when given early in the disease [45,46], with nearly 90% clinical efficacy [47–50]. However, despite early administration of drug, calves infected with resistant isolates showed more severe clinical outcomes, including prolonged fever and slower recovery of haematological parameters compared to the susceptible control group calves. Importantly, the parasitological findings clearly demonstrated the reduced efficacy of BPQ for resistant isolates with detection of parasites up to day 27. In contrast, previous *in vivo* studies reported that the majority of schizonts and piroplasms degenerate and become pycnotic within 24–48 hours of BPQ treatment, followed by a rapid decrease in parasitaemia to undetectable levels within 2–7 days [26,51–53].

Similar to the observations of Mhadhbi et al. (2010) [2], the untreated calves showed spontaneous recovery over time in the present study. Thus, it is likely that the recovery observed in the treated calves may be primarily due to their innate resistance to disease rather than the impact of treatment *per se* [54].

In order to investigate the premise that resistance is likely to be linked to the emergence of drug-resistant genotypes and the removal of drug-sensitive ones in a treated population *in vivo*, MTT colorimetric assays and AS-PCR were carried out on isolated cell lines. Consistent with earlier findings [6], cell lines from resistant groups (G2 and G3) displayed markedly reduced sensitivity to BPQ. Interestingly while resistance remained consistently high in lines representing animals in G2, a progressive increase in resistance was observed following successive treatments in G3 animals, suggesting an enrichment for parasite genotypes possessing a resistant phenotype. This difference and the variability in IC_50_ values between individual cell lines likely reflected the coexistence of resistant and susceptible parasite populations within the same host, a view supported by AS-PCR results [55–57]. Moreover, since the exact mechanism of BPQ resistance is not yet fully understood, the variation in IC_50_ values may arise from a mechanism similar to the bystander effect observed in cancer cells [58]. Thus, drug-resistant parasite populations might provide partial protection to a drug-susceptible population within the same *T. annulata* schizont-infected cell line. It is also possible that epigenetic events occur during the establishment of lines *in vitro* and these could theoretically influence susceptibility to the drug. Additionally, it should be noted that the MTT assay can be influenced by cellular factors such as cell population density, growth phase, and formazan extrusion, as well as non-cellular factors, including culture medium composition, tested treatments and formazan solvent agents [59]. Thus, the biological mechanisms underlying BPQ resistance of cell lines may be complex and could complicate conclusions that can be inferred from MTT assay data alone.

To evaluate the impact of BPQ treatment on allele frequencies within the parasite population, a set of chromosomal micro- and mini-satellite markers utilised previously [39,40] was employed. No evidence for selection of chromosomal genotype gain or loss was observed following repeated drug treatment of the *in vivo* infections. The reason for the apparent lack for emergence of a predominant resistant chromosomal genotype *in vivo* is unclear. It is important to note that the chromosomal markers used for population genetic structure analysis are physically unlinked to the candidate *Cyto b* loci within the mitochondrial genome. Satellite marker analysis revealed substantial calf-to-calf variation, particularly in G2 and G3 groups. While, none of the parasite populations from the experimental groups exhibited a significant shift in genotype turnover following drug treatment, it may be noteworthy that the population with the greatest level of drug resistance (G2 group) showed reduced heterozygosity. Also, some evidence of selection in the parasite population was observed at the level of the individual animal. A general turnover of genotypes was also noted in G1, even in the absence of selective pressure. The lack of evidence for genotypic selection linked to drug resistance has also been observed in the related apicomplexan parasite *P. falciparum* [60]. These results suggest that while drug treatment can select for resistant strains, it does not necessarily drive widespread genetic restructuring within the parasite population. Another inference is that BPQ resistance may be associated with a multilocus phenotype, with different parasite genotypes exhibiting resistance through distinct loci, such as *TaPIN1* previously linked to BPQ resistance [16] or different mutations in *Cyto b*. It is also known that BPQ treatment does not completely eliminate *Theileria* infections *in vivo* [26,61,62], suggesting that drug-sensitive parasite genotypes may persist in peripheral blood even after treatment, or that drug resistance is more common than thought previously.

Muraguri et al. (2006) [23] postulated that severe infections in animals that die, despite treatment, might affect the plasma distribution of BPQ due to the pathological disorder caused by theileriosis. However, our results demonstrated that the severity of theileriosis did not significantly affect the pharmacokinetics of BPQ in infected calves compared to uninfected ones. The findings were generally consistent with observations of animals mildly infected with *T. parva* [23] but show some noticeable differences in pharmacokinetic dynamics of BPQ compared with studies carried out by Kinabo and Bogan (1988) [22] and Muraguri et al. (2006) [23]. These differences may be attributed to factors such as variations in animal breed, age, weight, and infection status, as well as methodological differences between the studies. The results of the pharmacokinetic analysis also revealed that three administrations of BPQ at 48-hour intervals significantly increased plasma drug concentrations in infected animals for all groups although there was no correlation between plasma concentration parameters and clinical or parasitological findings. This agrees with previous studies demonstrating no association between plasma concentrations of parvaquone [63] and BPQ [23] and the treatment response in cattle infected with *T. parva.* However, the recovery of calves infected in G2 and G3 took longer, and these animals had a lower rate of dead parasites than animals in G1. Together, our results suggest that treatment failures among animals in groups 2 and 3 are not attributable to altered drug distribution but reflect intrinsic parasite resistance to BPQ.

A better understanding of how BPQ resistance impacts parasite fitness, in terms of its transmissibility, is a prerequisite for the future development of control methods against tropical theileriosis. Mhadhbi et al. (2010) [2] demonstrated that the BPQ-resistant *T. annulata* phenotype was transmissible through ticks. Two experiments performed in the present study demonstrated that resistant parasite populations harbouring mutations in *Cyto b* can be transferred between the host and vector ticks. However, detailed comparison revealed that the two experiments differed in terms of the obtained results. In the first experiment, ticks were fed on calves during the acute phase of theileriosis with a high level of parasitaemia, in order to prepare GUTS without any drug pressure. Parasites encoding V135A or P253S *Cyto b* mutations were transferred to vector ticks. This situation was also seen in our previous field study in Türkiye, in which carrier animals harboured both mutated (V135A and/or P253S) and unmutated parasite populations [6], indicating the persistence of the mutated parasite population in field. In the second experiment, the calves in G2 and G3 were infected with GUTS representing drug resistant parasite isolates and received repeated four BPQ treatments. After at least four months, without any drug pressure, they became carriers and ticks were fed on the calves. Only P253S mutants were transmitted to ticks from the calf from G3, with around 60% of the ticks testing positive. The reason for this differing transmission capability is not fully understood but may depend not on the abundance of parasites at the time of tick feeding or potential fitness costs associated with specific *Cyto b* (and other BPQ resistance linked) mutations.

Thus, given the level of drug-resistance determined for the G2 group was higher than G3 (Table 2), it may have a greater impact on fitness/transmissibility of that parasite population. Moreover, P253S linked to the G3 group appears to be the most geographically widespread BPQ resistance mutation, as reported in studies conducted in Tunisia [7], Iran [3], Egypt [5], Pakistan [64] and India [65]. This mutation, therefore, may have arisen multiple times independently or was the earliest and strongest to have arisen, subsequently spreading across endemic regions and conferring widespread emergence of BPQ-resistance. A recent study has shown that *Babesia microti* parasites, despite harbouring an A218V mutation in the *Cyto b* gene that is associated with a fitness cost, can be effectively transmitted from the vector tick to the host in response to drug treatment [66]. We conclude that similar to the A218V mutation in the *Cyto b* gene, the presence of P253S does not affect parasite transmission.

In conclusion, the present study provides evidence that resistant parasite populations with the V135A and P253S mutations are associated with more severe and persistent disease in treated hosts with high plasma concentrations of BPQ compared to parasites lacking these substitutions in the *Cyto b* gene. Furthermore, parasite populations harbouring these mutations can be transferred between the host and vector ticks. The extent to which mutations in the *Cyto b* gene affect the fitness of drug-resistant parasite genotypes and their transmission remains to be determined, but warrants further investigation, as it has implications for expansion of drug resistance in the field and control of the disease.

## Supporting information

S1 Table Experimental design and GUTS used for the infection. (PDF)

S2 Table Percentage of piroplasm parasitaemia before and after treatment in calves experimentally infected with susceptible (G1), and resistant (G2 and G3) GUTS. *: indicates the days when the calves were treated with BPQ.
(PDF)

S3 Table *T. annulata* schizont-infected cell lines isolated from calves in each group. BT: indicates before BPQ treatment, AT 1–4: indicate the number of repeated BPQ treatments. + : indicates successfully isolated schizont infected cell lines. NE: not established.
(PDF)

S4 Table AS-PCR results of *T. annulata* schizont-infected cell lines isolated from calves in each group and blood samples obtained before and after each treatment and on day 31 PI. (PDF)

S5 Table Alleles detected using five representative markers (TS5, TS20, TS25, TMSC75 and TMSC77) in parasite populations in G1 calves. BT: indicates before BPQ treatment. AT: indicates the number of repeated BPQ treatments. D31: indicates day 31 PI.
(PDF)

S6 Table Alleles detected using five representative markers (TS5, TS20, TS25, TMSC75 and TMSC77) in parasite populations in G2 calves. BT: indicates before BPQ treatment. AT: indicates the number of repeated BPQ treatments. D31: indicates day 31 PI.
(PDF)

S7 Table Alleles detected using five representative markers (TS5, TS20, TS25, TMSC75 and TMSC77) in parasite populations in G3 calves. BT: indicates before BPQ treatment. AT: indicates the number of repeated BPQ treatments. D31: indicates day 31 PI.
(PDF)
