# Supplementary material for: In vivo assessment of buparvaquone resistant Theileria annulata populations: genetic structure, transmission dynamics, drug susceptibility and pharmacokinetics
Source: PLoS One. 2025 Oct 15;20(10):e0334332. doi: 10.1371/journal.pone.0334332 (PMC12527135; doi:10.1371/journal.pone.0334332)
Supplement: S1 Table — (PDF) [file pone.0334332.s001.pdf]

S1 Table. Experimental design and GUTS used for the infection.

Groups	Calf ID (gender)	Weight (kg)	GUTS	Dose	Susceptibility or resistance / Mutation
**G1**	**1065** (Female)<br>**6859** (Female)<br>**9270** (Female)<br>**1344** (Male)	70<br>107.5<br>118<br>80.5	Ank/279	1.0 t.e.	Susceptible / none
**G2**	**2155** (Male)<br>**6857** (Female)<br>**1343** (Female)<br>**6816** (Female)	102<br>108<br>71<br>86.5	A10/BT	1.0 t.e.	Resistant / V135A
**G3**	**0770** (Male)<br>**6825** (Female)<br>**1135** (Female)<br>**3674** (Female)	104<br>81.5<br>105<br>101	A21/AT1	0.5 t.e.	Resistant / P253S
