# Supplementary material for: In vivo assessment of buparvaquone resistant Theileria annulata populations: genetic structure, transmission dynamics, drug susceptibility and pharmacokinetics
Source: PLoS One. 2025 Oct 15;20(10):e0334332. doi: 10.1371/journal.pone.0334332 (PMC12527135; doi:10.1371/journal.pone.0334332)
Supplement: S2 Table — *: indicates the days when the calves were treated with BPQ. (PDF) [file pone.0334332.s002.pdf]

S2 Table. Percentage of piroplasm parasitaemia before and after treatment in calves experimentally infected with susceptible (G1), and resistant (G2 and G3) GUTS

Experimental Groups	Days post-infection (% of live / degenerate piroplasm parasitaemia)																		
	Day 9	Day 10	Day 11	Day 12	Day 13	Day 14	Day 15	Day 16	Day 17	Day 18	Day 19	Day 20	Day 21	Day 22	Day 23	Day 24	Day 25	Day 27	Day 28
**G1**																			
1065	0.5*/0.0	0.4/0.2	0.3*/1.1	0.1/0.3	0.00*/0.00	0.10/0.66	0.16/0.58	0.12/0.53	0.00/0.51	0.00/0.50	0.00/0.34	0.00/0.00	0.00/0.31	0.08/0.31	0.07/0.26	0.00/0.35	0.16/0.40	0.26/0.09	0.34/0.17
9270	0.34*/0.0	0.18/0.36	0.36*/0.18	0.00/0.50	0.00*/0.00	0.00/0.00	0.00*/0.00	0.00/0.00	0.00/0.00	0.00/0.00	0.00/0.00	0.00/0.00	0.00/0.00	0.00/0.00	0.00/0.00	0.00/0.00	0.00/0.00	0.00/0.00	0.00/0.00
6859	0.45*/0.0	0.30/0.30	0.42*/0.16	0.15/0.59	0.00*/0.00	0.05/0.74	0.04*/0.71	0.00/0.66	0.00/0.71	0.00/0.35	0.00/0.28	0.00/0.42	0.00/0.43	0.00/0.36	0.00/0.29	0.00/0.31	0.00/0.24	0.00/0.00	0.00/0.00
1344	0.34*/0.0	0.06/0.24	0.18*/0.06	0.16/0.49	0.16*/0.00	0.00/0.00	0.00*/0.76	0.00/0.00	0.00/0.50	0.00/0.27	0.00/0.00	0.00/0.00	0.00/0.00	0.00/0.00	0.00/0.00	0.00/0.00	0.00/0.00	0.00/0.00	0.00/0.00
**G2**																			
6816	0.00/0.00	0.00/0.00	1.68*/0.06	3.45/0.71	15.17*/1.36	12.55/2.35	10.38*/3.66	17.80/2.20	11.81*/6.00	7.55/4.51	7.94/2.81	8.45/4.73	7.01/5.51	5.42/5.88	5.61/4.87	6.69/4.34	5.56/2.73	3.90/1.45	0.46/0.92
1343	0.00/0.00	0.00/0.00	0.45*/0.05	1.37/0.28	15.58*/0.53	9.46/0.90	10.49*/1.72	11.72/1.49	10.39*/4.63	8.05/5.17	9.39/3.83	7.92/3.44	6.64/3.10	4.77/2.26	3.08/1.68	2.87/1.49	1.29/0.52	0.52/0.41	0.28/0.28
2155	0.00/0.00	0.00/0.00	0.58*/0.06	0.36/0.18	1.06*/0.23	0.89/0.33	1.34*/0.32	0.93/0.16	0.65*/0.46	0.42/0.32	0.53/0.42	0.47/0.23	0.26/0.18	0.15/0.23	0.00/0.00	0.00/0.20	0.00/0.24	0.21/0.21	0.00/0.00
6857	0.00/0.00	0.00/0.00	0.38*/0.00	0.48/0.07	1.71*/0.24	1.64/0.52	2.74*/0.57	2.38/0.48	1.68*/0.66	0.70/0.60	0.27/0.46	0.21/0.54	0.31/0.10	0.11/0.42	0.11/0.22	0.00/0.00	0.00/0.00	0.00/0.00	0.00/0.00
**G3**																			
0770	0.00/0.00	0.00/0.0	0.96*/0.00	0.71/0.27	0.63 */0.43	0.62/0.35	0.63 */0.38	0.91 /0.21	0.55*/0.28	0.16/0.28	0.16/0.32	0.21/0.21	0.21/0.35	0.25/0.25	0.15/0.23	0.27/0.33	0.26/0.20	0.07/0.30	0.06/0.13
6825	0.00/0.00	0.00/0.0	0.58*/0.00	0.16/0.38	0.57 */0.40	0.94 /0.22	1.45 */0.08	1.05/ 0.21	0.39*/ 0.22	0.00/0.30	0.00/0.36	EX	-	-	-	-	-	-	-
3674	0.00/0.00	0.00 /0.0	0.70*/0.00	0.20/0.29	0.70* /0.35	0.85/ 0.28	1.26*/0.26	0.78/ 0.17	0.38*/ 0.23	0.08/0.30	0.00/0.37	0.00/0.28	0.00/0.28	0.00/0.29	0.00/0.35	0.00/0.31	0.18/0.27	0.00/0.38	0.00/0.00
1135	0.00/0.00	0.43*/0.0	0.47/0.00	0.35*/0.18	1.22 /0.33	1.82*/0.42	1.88 /1.65	1.95*/0.42	0.93/ 0.60	1.31/0.93	1.52/0.91	1.17/0.68	0.88/0.81	1.24/0.85	2.14/0.97	1.68/0.95	2.02/1.05	2.72/1.15	0.98/0.53

*: indicates the days when the calves were treated with BPQ
