# Supplementary material for: In vivo assessment of buparvaquone resistant Theileria annulata populations: genetic structure, transmission dynamics, drug susceptibility and pharmacokinetics
Source: PLoS One. 2025 Oct 15;20(10):e0334332. doi: 10.1371/journal.pone.0334332 (PMC12527135; doi:10.1371/journal.pone.0334332)
Supplement: S3 Table — BT: indicates before BPQ treatment, AT 1–4: indicate the number of repeated BPQ treatments. + : indicates successfully isolated schizont infected cell lines. NE: not established. (PDF) [file pone.0334332.s003.pdf]

S3 Table. *T. annulata* schizont-infected cell lines isolated from calves in each group

Experimental Groups	BT	AT 1	AT 2	AT 3	AT4	DAY 31
**G1**						
1065	+	+	+			
9270	+	+	+			
6859	+	+	NE			
1344	+	+	+	+		
**G2**						
1343	+	NE	+	+	+	+
6857	+	+	+	+	+	+
2155	+	+	+	+	+	
6816	+	+	+	+	+	+
**G3**						
3674	+	+	+	+	+	
1135	+	+	+	+	+	
6825	+	NE	+	+	+	
0770	+	+	NE	+	+	

BT: indicates before BPQ treatment
AT 1-4: indicate the number of repeated BPQ treatments.
+: indicates successfully isolated schizont infected cell lines.
NE: not established
