# Supplementary material for: In vivo assessment of buparvaquone resistant Theileria annulata populations: genetic structure, transmission dynamics, drug susceptibility and pharmacokinetics
Source: PLoS One. 2025 Oct 15;20(10):e0334332. doi: 10.1371/journal.pone.0334332 (PMC12527135; doi:10.1371/journal.pone.0334332)
Supplement: S4 Table — (PDF) [file pone.0334332.s004.pdf]

S4 Table. AS-PCR results of *T. annulata* schizont-infected cell lines isolated from calves in each group and blood samples obtained before and after each treatment and on day 31 PI.

Experimental Groups	Presence of mutations in cell lines / blood samples																	
	BT			AT1			AT2			AT3			AT4			31st day of experiment		
	V135A	P253S	Wild Type	V135A	P253S	Wild Type	V135A	P253S	Wild Type	V135A	P253S	Wild Type	V135A	P253S	Wild Type	V135A	P253S	Wild Type
**G1**																		
1065	-/-	-/-	+/+	-/-	-/-	+/+	-/-	-/-	+/+	-/-	-/-	+/+	-/-	-/-	+/+	-/-	-/-	+/+
9270	-/-	-/-	+/+	-/-	-/-	+/+	-/-	-/-	+/+	-/-	-/-	+/+	-/-	-/-	+/+	-/-	-/-	+/+
6859	-/-	-/-	+/+	-/-	-/-	+/+	-/-	-/-	+/+	-/-	-/-	+/+	-/-	-/-	+/+	-/-	-/-	+/+
1344	-/-	-/-	+/+	-/-	-/-	+/+	-/-	-/-	+/+	-/-	-/-	+/+	-/-	-/-	+/+	-/-	-/-	+/+
**G2**																		
1343	+/+	-/-	+/+	+	-/-	+/+	+/+	-/-	+/+	+/+	-/-	+/+	+/+	-/-	+/+	+/+	-/-	+/+
6857	+/+	-/-	+/+	+	-/-	+/+	+/+	-/-	+/+	+/+	-/-	+/+	+/+	-/-	**-/+**	+/+	-/-	**-/+**
2155	+/+	-/-	+/+	+	-/-	+/+	+/+	-/-	+/+	+/+	-/-	+/+	+/+	-/-	**-/+**	+/+	-/-	**-/+**
6816	+/+	-/-	+/+	+	-/-	+/+	+/+	-/-	+/+	+/+	-/-	+/+	+/+	-/-	+/+	+/+	-/-	+/+
**G3**																		
3674	-/-	+/+	+/+	-/-	+/+	+/+	-/-	+/+	+/+	-/-	+/+	+/+	-/-	+/+	+/+	-/-	+/+	+/+
1135	-/-	**-/+**	+/+	-/-	+/+	+/+	-/-	+/+	+/+	-/-	+/+	+/+	-/-	+/+	+/+	-/-	+/+	+/+
6825	-/-	+/+	+/+	-/-	+/+	+/+	-/-	+/+	+/+	-/-	+/+	+/+	-/-	+/+	+/+	-/-	+/+	+/+
0770	-/-	+/+	+/+	-/-	+/+	+/+	-/-	+/+	+/+	-/-	+/+	+/+	-/-	+/+	+/+	-/-	+/+	+/+
