# Supplementary material for: In vivo assessment of buparvaquone resistant Theileria annulata populations: genetic structure, transmission dynamics, drug susceptibility and pharmacokinetics
Source: PLoS One. 2025 Oct 15;20(10):e0334332. doi: 10.1371/journal.pone.0334332 (PMC12527135; doi:10.1371/journal.pone.0334332)
Supplement: S5 Table — BT: indicates before BPQ treatment. AT: indicates the number of repeated BPQ treatments. D31: indicates day 31 PI. (PDF) [file pone.0334332.s005.pdf]

S5 Table. Alleles detected using five representative markers (TS5, TS20, TS25, TMSC75 and TMSC77) in parasite populations in G1 calves

Marker	Treatment status		G1 calves			
			**1065**	**9270**	**6859**	**1344**
**TS5**	**BT**		0	0	0	268-278
	**AT**	**1**	268-278	0	0	0
		**2**	0	0	268-278	268-278
		**3**	0	0	268-278	268-278
		**4**	0	268-278	0	0
	**D31**		268-278	0	268-278	268-278
**TS20**	**BT**		234-252-354	234-252-354	234-252-305-354	234-252-354
	**AT**	**1**	234-252-354	234-252-354	234-252-354	234-252-354
		**2**	234-252-354	0	234-252-354	234-252-354
		**3**	234-252	234-252	234-252-354	234-252-354
		**4**	234-252-354	252	234-252-354	252
	**D31**		234-252-354	252	252	234-252-354
**TS25**	**BT**		232-252-346	232-252-346	252-346	252-346
	**AT**	**1**	232-248-252-311-346	232-248-252-311-346	232-248-252-311-346	232-248-252-311-346
		**2**	232-248-252-311-346	0	232-248-252-311-346	232-248-252-311-346
		**3**	232-248-252	232-248-252	232-248-252-311-346	232-248-252-311-346
		**4**	232-252-346	232-248-252	232-248-252-346	232-248-252
	**D31**		232-248-252-311-346	248	232-248-252	232-248-252-346

**TMSC75**	**BT**		204-230-240	204-230-240	204-230-240	204-230-240
	**AT**		204-230-240-278	204-230-240-278	204-230-240	204-230-240-278
			204-230-240-278	204-230-240	204-230-240	204-230-240-278
			204-230-240-278	204-230-240-278	204-230-240-278	204-230-240-278
			204-230-240	204-230-240	204-230-240	204-230-240
	**D31**		204-230-240-278	204-230-240-278	204-230-240-278	204-230-240-278
**TMSC77**	**BT**		189-208-223-285	189-208-223-285	189-208-223-285	189-208-223-285
	**AT**	**1**	189-208-223-285	189-208-223-285	189-208-223-285	189-208-223-285
		**2**	189-208-223-285	189-208-223	189-208-223-285	189-208-223-285
		**3**	189-208-223-285	189-208-223-285	189-208-223-285	189-208-223-285
		**4**	189-208-223-285	189-208-223	189-208-223-285	189-208-223
	**D31**		189-208-223-285	189-208-223-285	189-208-223-285	189-208-223-285

BT: indicates before BPQ treatment

AT: indicates the number of repeated BPQ treatments

D31: indicates day 31 post infection
