# Supplementary material for: In vivo assessment of buparvaquone resistant Theileria annulata populations: genetic structure, transmission dynamics, drug susceptibility and pharmacokinetics
Source: PLoS One. 2025 Oct 15;20(10):e0334332. doi: 10.1371/journal.pone.0334332 (PMC12527135; doi:10.1371/journal.pone.0334332)
Supplement: S6 Table — BT: indicates before BPQ treatment. AT: indicates the number of repeated BPQ treatments. D31: indicates day 31 PI. (PDF) [file pone.0334332.s006.pdf]

S6 Table. Alleles detected using five representative markers (TS5, TS20, TS25, TMSC75 and TMSC77) in parasite populations in G2 calves

Marker	Treatment status		G2 calves			
			6816	1343	2155	6857
TS5	BT		242-244	242-244	244	242-244
	AT	1	242-244-268-295	242-244-268-295	242-244-268-295	242-244-268-295
		2	242-244-295	0	0	0
		3	242-244-268-295	242-244-268-295	242-244-268-295	242-244-268-295
		4	242-244-268-295	242-244-268-295	242-244-268-295	264
	D31		242-244-268-295	244-268-290	244-268	0
TS20	BT		232	232	232	232
	AT	1	232	232	232	232
		2	232	232	232	232
		3	232	232	232	232
		4	232	232	232	0
	D31		232	0	232	0
TS25	BT		234-238	234-238	234-238	234-238
	AT	1	234-238	234-238	234-238	234-238
		2	234-238	234-238	234-238	234-238
		3	234-238	234-238	234-238	234-238
		4	234-238	0	234-238	234-238
	D31		234-238	0	234-238	0

**TMSC75**	**BT**		232-236	232-236-258-260	232-236	232-236
	**AT**		232-236-258-260	232-236-258-260	232-236-258-260	232-236-258-260
			232-236-258-260	232-236-258-260	232-236-258-260	232-236-258-260
			232-236-258-260	232-236-258-260	232-236-258	232-236-258-260
			232-236-258-260	206-230-232-242	232-236-258-260	232-236-258-260
	**D31**		232-236-258	236-240	232-236-258	232-236
**TMSC77**	**BT**		200	200	200	200
	**AT**	**1**	200	200	200	200
		**2**	200	200	200	200
		**3**	200	200	200	200
		**4**	200	200	200	200
	**D31**		200	200	200	200

BT: indicates before BPQ treatment

AT: indicates the number of repeated BPQ treatments

D31: indicates day 31 post infection
