# Supplementary material for: In vivo assessment of buparvaquone resistant Theileria annulata populations: genetic structure, transmission dynamics, drug susceptibility and pharmacokinetics
Source: PLoS One. 2025 Oct 15;20(10):e0334332. doi: 10.1371/journal.pone.0334332 (PMC12527135; doi:10.1371/journal.pone.0334332)
Supplement: S7 Table — BT: indicates before BPQ treatment. AT: indicates the number of repeated BPQ treatments. D31: indicates day 31 PI. (PDF) [file pone.0334332.s007.pdf]

S7 Table. Alleles detected using five representative markers (TS5, TS20, TS25, TMSC75 and TMSC77) in parasite populations in G3 calves

**Marker**	**Treatment status**		**G3 calves**			
			**0770**	**6825**	**3674**	**1135**
**TS5**	**BT**		238-255-258-268-288	238-255-258-268-288	238-255-258-268-288	0
	**AT**	**1**	238-255-258-268-288	238-255-258-268-288	238-255-258-268-288	255-268-285-288
		**2**	238-255-258-268-285-288	238-255-258-268-285-288	238-255-258-268-285-288	255-268-285-288
		**3**	238-255-258-268-285-288	238-255-258-268	238-255	255-268-285-288
		**4**	0	238-255-258-268-288	238-255-258-268	0
	**D31**		258-270	238-255-258-268-288	0	258-270-285-288
**TS20**	**BT**		266-269-342	232-269-342	269	232
	**AT**	**1**	266-269-342	266-269-342	266-269-342	266-269-342
		**2**	266-269-292-342	266-269-292-342	232-266-269-292	266-269-342
		**3**	266-269-292-342	266-269-292-342	266-269-292-342	266-269-292-342
		**4**	269-292	266-269-292-342	269-292	269-292
	**D31**		229-269-301	232-269-342	229-269	269-292
**TS25**	**BT**		216-224-236-267	216-224-236-267	216-224-236	219-232
	**AT**	**1**	216-224-236-267	216-224-236-267	216-224-236-267	216-224-236-267
		**2**	216-224-236-267	216-224-236-267	216-224-236	216-224-236-267
		**3**	216-224-236-267	216-224-236-267	216-224-236	216-224-236-267
		**4**	216-224-236-267	216-224-236-267	216-224-236-267	216-224-236-267
	**D31**		229-236	216-224-236-267	229-236	216-224-236

**TMSC75**	**BT**		206-236-240	206-236-240	206-236-240	236-240-258
	**AT**		206-236-240	206-236-240	236-240	206-236-240
			206-236-240	206-236-240	236-240	206-236-240
			206-236-240	206-236-240	236-240	206-236-240
			206-236-240	206-236-240	236-240	206-236-240
	**D31**		236-240	206-236-240	236-240	236-240
**TMSC77**	**BT**		204-215-220	204-215-220	204-215-220	204-215-220
	**AT**	**1**	204-215-220	204-215-220	204-215-220	204-220
		**2**	204-215-220	204-220	204-215-220	204-215-220
		**3**	204-215-220	204-220	204-215-220	204-215-220
		**4**	204-215-220	204-215-220	204-215-220	204-215-220
	**D31**		204-215-220	204-215-220	204-215-220	204-220

BT: indicates before BPQ treatment

AT: indicates the number of repeated BPQ treatments.

D31: indicates day 31 post infection
